# A Comparative Study of Information-Based Source Number Estimation Methods and Experimental Validations on Mechanical Systems

**DOI:** 10.3390/s140507625

**Published:** 2014-04-25

**Authors:** Wei Cheng, Zhousuo Zhang, Hongrui Cao, Zhengjia He, Guanwen Zhu

**Affiliations:** State Key Laboratory for Manufacturing Systems Engineering, Xi'an Jiaotong University, Xi'an 710049, China; E-Mails: zzs@mail.xjtu.edu.cn (Z. Z.); chr@mail.xjtu.edu.cn (H.C.); hzj@mail.xjtu.edu.cn (Z.H.); zhuguanwen@gmail.com (G.Z.)

**Keywords:** source number estimation, Akaike information criterion, minimum description length, improved Bayesian information criterion, eigenvalue decomposition

## Abstract

This paper investigates one eigenvalue decomposition-based source number estimation method, and three information-based source number estimation methods, namely the Akaike Information Criterion (AIC), Minimum Description Length (MDL) and Bayesian Information Criterion (BIC), and improves BIC as Improved BIC (IBIC) to make it more efficient and easier for calculation. The performances of the abovementioned source number estimation methods are studied comparatively with numerical case studies, which contain a linear superposition case and a both linear superposition and nonlinear modulation mixing case. A test bed with three sound sources is constructed to test the performances of these methods on mechanical systems, and source separation is carried out to validate the effectiveness of the experimental studies. This work can benefit model order selection, complexity analysis of a system, and applications of source separation to mechanical systems for condition monitoring and fault diagnosis purposes.

## 1. Introduction

In many physical systems, the measured signals can be modeled as a superposition of a finite number of the sources with additive environmental noises, and many signal processing methods such as principal component analysis (PCA) [1], blind source separation (BSS) [2,3] and independent component analysis (ICA) [4–6] have benefited from this model and achieved wide use in engineering applications. A key and primary issue of these subjects is the estimation of the number of unknown sources from the mixed signals before an effective source separation. Furthermore, it is still challenging to estimate the source number for mechanical systems due to the complicated mixing of the sources and the transmission effects of the mechanical structures.

In the past decades, many researchers have focused their interests on source number estimation methods and their engineering applications, and proposed many approaches to solve this problem. Ye *et al.* [7] studied the general BSS problem satisfying *m* greater than or equal to *n* and gave the validations by computer simulations on artificially synthesized data. Fishler *et al.* [8] studied a MDL-type estimator that was robust against deviation from the assumption of equal noise level across the array. Bai *et al.* [9] proposed an information-based method to estimate the number of independent dipole sources from electroencephalograms (EEGs). Jiang *et al.* [10] proposed a new source number estimation method called beam eigenvalue method (BEM). Huang *et al.* [11,12] proposed to utilize the minimum mean square error (MMSE) of the multistage Wiener filter to calculate the required description length for encoding the observed data, instead of relying on the eigenvalues of the data covariance matrix. Hu *et al.* [13] proposed a sound source number and directions estimation method under a multisource reverberant environment and gave experimental validations. Ma *et al.* [14] proposed a source number estimation method based on modified K-means clustering. Cheng *et al.* [15] proposed an independent component analysis-based source number estimation methods and applied it to mechanical systems. Han *et al.* [16] proposed a source number estimation method based on uniform linear arrays (ULAs) and the newly proposed nested arrays. Dosso *et al.* [17] considered localizing an unknown number of ocean acoustic sources when the properties of the environment are poorly known. Sadhu *et al.* [18] proposed a decentralized model identification method utilizing the concepts of sparse blind source separation and parallel factor decomposition, which can solve underdetermined blind source separation problems. All the above studies have provided effective ways to estimate the number of sources from different types of the sources. However, the studies on the source number estimation for mechanical systems are very few, and there is still a lot of work to do before an effective application of source number estimation methods to mechanical systems can be put forth.

Unlike the eigenvalue decomposition-based source number estimation method which requires a threshold, information criteria-based methods do not need any parameters for adaptively estimating the number of sources from the mixed signals, and the algorithms are also easier for calculation and perform efficiently in the applications. The key issue on the information-based methods is to find the extremum values of the constructed objective functions based on information criteria, such as Akaike Information Criterion (AIC) [19,20], Minimum Description Length (MDL) [21–23], and Bayesian Information Criterion (BIC) [24,25]. However, it has been shown that AIC suffers from the computational problem [26]. MDL also suffers from high computational load and performs well only in the presence of spatially and temporally white noise [27,28]. Furthermore, they have a shortcoming in that the estimation performance is sensitive to the signal to noise ratio (SNR) and the sampling length, and thus the results may be unreliable. Studies on the source number estimation by BIC are rarely found, and the traditional BIC will overflow in the calculation if the sampling length is too large. Furthermore, all the three information-based methods are rarely applied to estimate the source number of mechanical systems whose sources are normally mixed according to linear superposition and nonlinear modulation.

Therefore, this paper studies comparatively the performances of both eigenvalue decomposition-based and information-based source number estimation methods on mechanical sound signals, and improves BIC as IBIC to make it easier for calculation and efficient for the data with a large sampling length. Both linear superposition and nonlinear modulation are considered in the numerical case studies, and a test-bed with three sources is constructed to test the performances of the eigenvalue decomposition-based and information-based methods on source number estimation for mechanical systems. This study can benefit for the model order selection, complexity analysis of a system, and applications of source separation to mechanical systems for condition monitoring and fault diagnosis purposes.

The remainder of this paper is organized as follows: in Section 2, we introduce the theoretical background and investigate the mathematical mechanisms of the eigenvalue decomposition-based source number estimation method, and information-based source number estimation methods entitled as AIC, MDL, and IBIC. In Section 3, we test the performances of these methods on typical mechanical signals with both a linear superposition and a nonlinear modulation. In Section 4, a test bed with three sound sources is constructed to further test the performances of these methods on real mechanical systems, and the effectiveness of the experimental studies is validated by source separation and spectral analysis. Finally, Section 5 summarizes the conclusions.

## 2. Source Number Estimation Methods

Consider *m* observed mixed signals **X**(*t*) = [**x**_1_(*t*), ⋯, **x**_m_(*t*)]*^T^* measured in different locations and composed by *n* source signals **S**(*t*) = [**s**_1_(*t*), ⋯, **s**_n_(*t*)]*^T^*. Assume that the observed signals can be described by the following model with white noises of **N**(*t*) = **n**_1_(*t*), **n**_2_(*t*), ⋯, **n**_m_(*t*) and mixing matrix of **A** = {a_ij_}_m×n_:

(1) $$\begin{array}{ll} x_{i}(t)=\overset{n}{\underset{j=1}{\text{∑}}}a_{ij}s_{j}(t)+n_{i}(t) & i=1,\cdots ,m,j=1,\cdots ,n\\ X(t)=AS(t)+N(t) & \left(\text{in a matrix form}\right) \end{array}$$

As the source signals and mixing mode are normally unknown for many physical systems, a crucial problem associated with this model is to estimate the number *n* of source signals from an *N* finite set of observations **x**_1_(*t*), ⋯, **x**_m_(*t*) before an effective source separation.

Constitute **X** = [**X**(t_1_), ⋯, **X**(t_N_)] from an *N* finite set of observations. Then the covariance matrix of **X**(·) is given by **R** = E[**XX***^T^*] (E[] is the expected function). Denoting the eigenvalues of **R** by λ_1_ ≥ λ_2_ ≥⋯ λ_m_, L(n) which is a log-likelihood function used to estimate the maximum likelihood of source number *n* is defined as follows:

(2) $$L(n)=\frac{{\left(\lambda_{n+1}\lambda_{n+2}\cdots \lambda_{m}\right)}^{\frac{1}{m-n}}}{\frac{1}{m-1}\left(\lambda_{n+1}+\lambda_{n+2}+\cdots +\lambda_{m}\right)}$$

(1) Source number estimation based on eigenvalue decomposition:
  (3) $$n^{*}=\underset{n}{\mathrm{argmax}}\left[\lambda_{n}\geq \gamma \right],n=1,2,\cdots ,m$$
  Where *γ* is a threshold.
  The benefit of eigenvalue decomposition is that the source number can be estimated just based on the distributions of eigenvalues, and the crucial step is just a reasonable threshold *γ*. However, different types of mixed signals have different distributions of eigenvalues, which makes it impossible for a threshold *γ* for all the applications.
  Now we comparatively introduce and investigate another three information-based source number estimation methods which can determine the source number adaptively.
(2) Akaike Information Criterion (AIC)
  The information theoretic criterion for the model order selection or source number estimation, introduced by Akaike [19] is used to determine the number of signals which gives the minimum AIC, defined by:
  (4) $$n^{*}=\underset{n}{\arg\min}\left[\mathrm{AIC}(n)\right]=\underset{n}{\arg\min}\left[-2N(m-n)\mathrm{lg}L(n)+2n(2m-n)\right]$$
  The first term, −2*N*(*m*−*n*)lgL(*n*), is the well-known log-likelihood of the maximum likelihood estimator of the parameters of the model. The second term, 2*n*(2*m*−*n*), is the bias correction term inserted so as to make AIC an unbiased estimator.
(3) Minimum Description Length (MDL) [19]
  Inspired by Akaike's work, Rissanen [21] proposed a different approach to select the model or estimate the source number based on the concept of the shortest code length for the data. It can be achieved by the following criterion:
  (5) $$n^{*}=\underset{n}{\arg\min}\left(\mathrm{MDL}(n)\right)=\underset{n}{\arg\min}\left(-N(m-n)\mathrm{lg}L(n)+\frac{1}{2}n(2m-n)\mathrm{lg}N\right)$$
  Note that apart from a factor of 2, the first term is identical to the corresponding one in AIC, while the second term has an extra factor of ½ lg*N*.
(4) Bayesian information criterion (BIC) [29]
  Minka [24] proposed another criterion for estimation of the dimensionality of the data (source number) on basis of Bayesian model selection. It is referred to as the Minka Bayesian model selection (MIBS) which is given by (assume that *σ̃* is the positive scale parameter, *λ* is the eigenvalue of the covariance matrix of mixed signals **X**, *m* is the number of mixed signals **X**, *n* is the number of sources **S**, and *N* is the data length):
  (6) $$\mathrm{MIBS}(n)\approx p_{n}{\left(\prod_{j=1}^{n}\lambda_{j}\right)}^{-N/2}{\tilde{\sigma }}_{n}^{-N(m-n)}{\left|A_{n}\right|}^{-1/2}{\left(2\pi \right)}^{\left(d_{n}+n\right)/2}N^{-n/2}$$
  where:
  (7) $$\begin{array}{l} p_{n}=2^{-n}\overset{n}{\underset{i=1}{\text{∏}}}\Gamma \left(\frac{m-i+1}{2}\right)\pi^{-\left(m-i+1\right)/2}\\ \left|A_{n}\right|=\overset{n}{\underset{i=1}{\text{∏}}}\overset{m}{\underset{j=i+1}{\text{∏}}}\left({\hat{\lambda }}_{j}^{-1}-{\hat{\lambda }}_{i}^{-1}\right)\left(\lambda_{i}-\lambda_{j}\right)N\\ {\hat{\sigma }}_{n}^{2}=\left(\overset{m}{\underset{j=n+1}{\text{∑}}}\lambda_{j}\right)/m-n\\ d_{n}=mn-n\left(n+1\right)/2 \end{array}$$
  and *λ̂_J_* is identical with *λ*_j_ except for *j* > *n* where ${\hat{\lambda }}_{J}=\hat{\sigma_{n}}$. In order to estimate the latent dimensionality of the data (source number), we choose the value *n* that maximizes Equation (5). The simplification of MIBS is the BIC approximation, which drops all terms that do not grow with *N*:
  (8) $$\mathrm{BIC}(n)={\left(\prod_{j=1}^{n}\lambda_{j}\right)}^{-N/2}{\tilde{\sigma }}_{n}^{-N(m-n)/2}N^{-\left(d_{n}+n\right)/2}$$
  In practice, it causes overflow in calculating Equation (7) when the value of *N* is large (Normally *N* is a big number). Therefore, we take the logarithm to overcome the overflow problem and entitle it as the improved BIC (IBIC), which significantly decreases the calculating time without a loss of the accuracy. The objective function of the IBIC is as follow equation:
  (9) $$\begin{array}{ll} n^{*} & =\underset{n}{\arg\max}\left(\mathrm{IBIC}(n)\right)=\underset{n}{\arg\max}\left(\mathrm{lg}\left(\mathrm{BIC}(n)\right)\right)\\ & =\underset{n}{\arg\max}\left(-\frac{N}{2}\mathrm{lg}\left(\overset{n}{\underset{j=1}{\text{∏}}}\lambda_{j}\right)-\frac{N(m-n)}{2}\mathrm{lg}\left({\tilde{\sigma }}_{n}\right)-\frac{\left(d_{n}+n\right)}{2}\;\mathrm{lg}\left(N\right)\right) \end{array}$$

## 3. Numerical Case Study

In this section, we numerically generate typical signals of mechanical systems to comparatively study the effectiveness of the different source number estimation methods. These generated source signals consider the modulation effects of mechanical systems, and the mixed signals are composed of the sources through a linear superposition and a weak nonlinear mixing. The generating functions of the source signals are listed below:

(10) $$S(t)=\left[\begin{array}{l} s_{1}(t)\\ s_{2}(t)\\ s_{3}(t)\\ s_{4}(t) \end{array}\right]=\left[\begin{array}{l} 3\times \sin(10\pi t)\\ 5\times \sin(4\times \cos(8\pi t))\\ 7\times \sin(5\pi t)\cos(80\pi t)\\ 9\times n(t) \end{array}\right]$$

**Figure 1.**
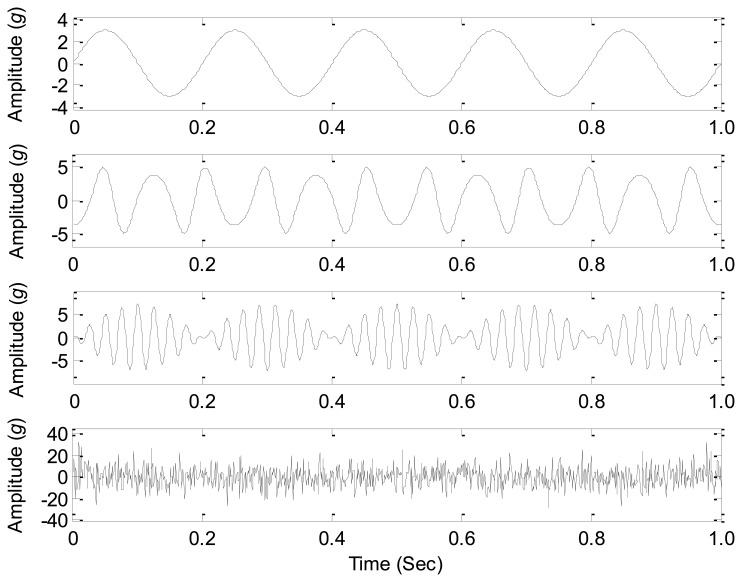
The waveforms of the source signals.

In the numerical case study, **s**_1_(*t*) is a sinusoidal signal that simulates the simple harmonic vibration of mechanical systems; **s**_2_(*t*) is a frequency modulation signal that simulates the frequency modulation effects of mechanical systems; **s**_3_(*t*) is an amplitude modulation signal that simulates the amplitude modulation effects of mechanical systems; **s**_4_(*t*) is a white noise signal that simulates the noises produced by the structural transmission and environment. The waveforms of the source signals are shown in Figure 1.

### 3.1. Source Number Estimation for Linearly Mixed Signals

Since the number of the mixed signals should be no less than the number of the source signals for an accurate source separation or system identification, and the source number estimation methods based on the information criteria also require more mixed signals, in the numerical case study we provide six mixed signals composed by the given source signals with a linear superposition matrix **A** given by:

$$A=\left[\begin{array}{cccc} 0.58 & 0.36 & -0.29 & 0.89\\ 0.33 & -0.65 & 0.49 & -0.93\\ 0.77 & 0.83 & -0.72 & -0.85\\ 0.18 & 0.51 & 0.83 & 0.79\\ 0.25 & -0.42 & 0.65 & -0.59\\ 0.43 & -0.27 & -0.14 & 0.32 \end{array}\right]$$

**Figure 2.**
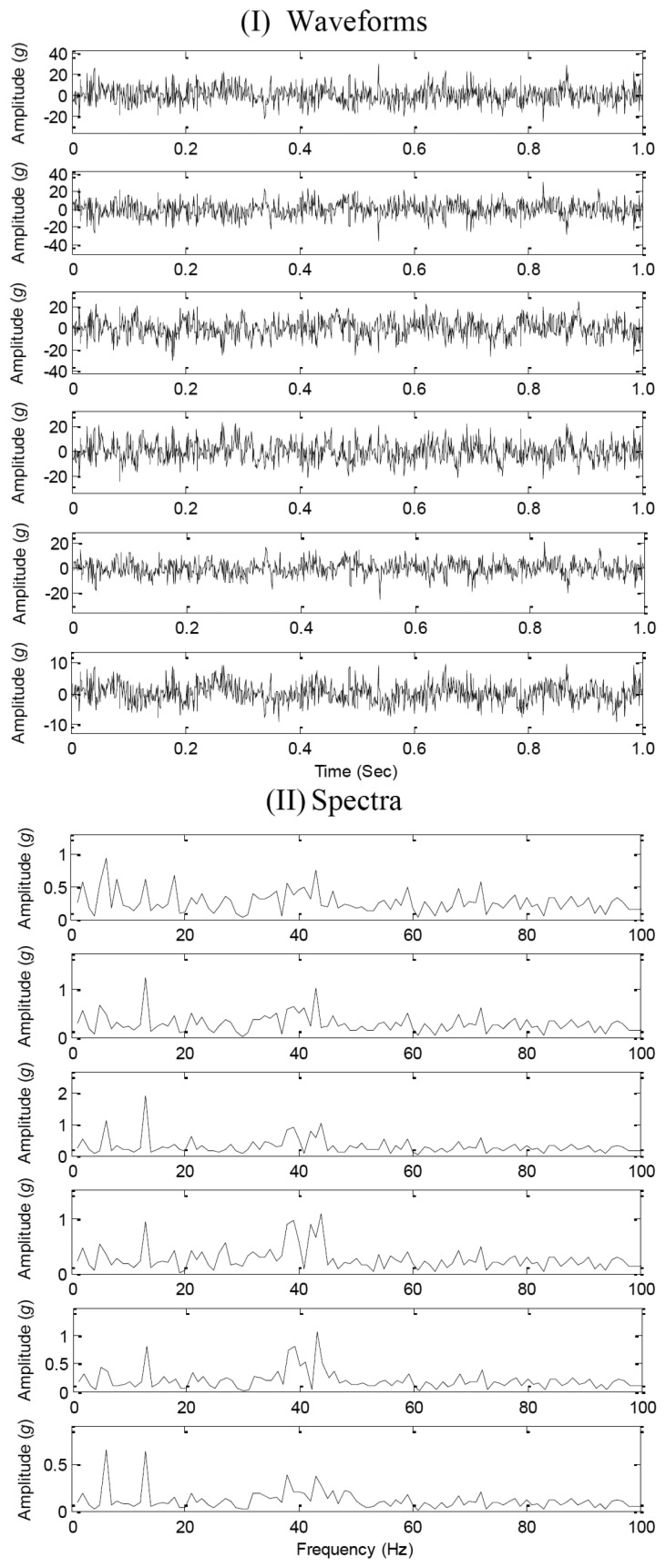
The waveforms and spectra of the mixed signals.

Figure 2 displays the waveforms and spectra of the mixed signals, which indicates that it is a difficult task to directly estimate the source number for complex waveforms and complex Fourier spectra with many major components. Therefore, source number estimation methods are required to reveal the complexity of the mixed signals.

Table 1 lists the eigenvalues' distribution of the covariance matrix for the mixed signals. Obviously eigenvalues decrease significantly from *λ*_1_ to *λ*_5_, even *λ*_5_ = *λ*_6_ = 0, which means that there are 4 principal components contained in the mixed signals (from the definition of principal component analysis [1]). Therefore, the threshold *γ* can be determined as *γ* ∈ (0, 4.42) and thus there has n* = 4. The result also shows that the threshold *γ* can be easily determined and the source number estimation based on eigenvalue decomposition is effective for the given linear superposition case.

**Figure 3.**
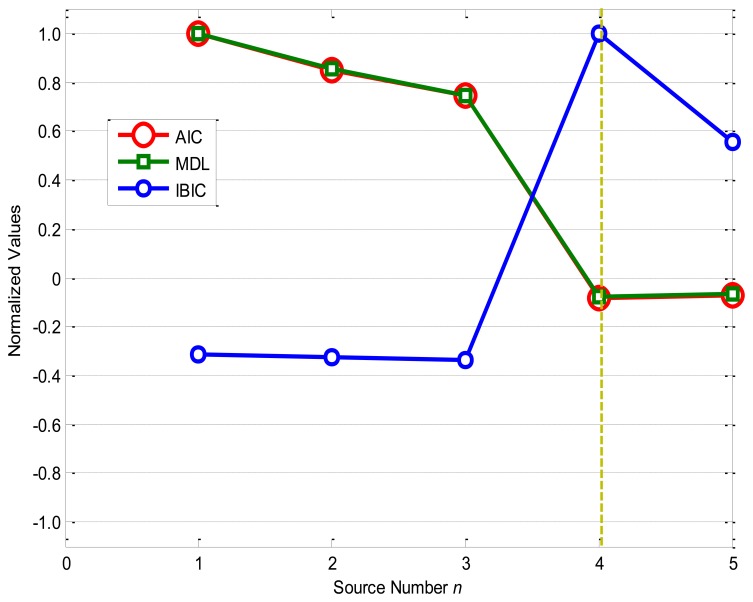
Source number estimation by information-based methods.

Figure 3 shows the source number estimation by the information-based source number estimation methods: as the source number *n* increases, the normalized objective functions of AIC and MDL decrease fast from *n* = 1 to *n* = 4, and obtain the minimum values −0.0809 and −0.0772 as *n* = 4, while the normalized objective function of IBIC changes greatly from *n* = 3 to *n* = 5, and obtains the maximum value 1.0000 as *n* = 4. From the definitions of the information-based methods, all these methods accurately evaluate the source number n* = 4 for the given numerical case with the linear superposition. It should be noted that AIC and MDL obtain very similar results from *n* = 1 to *n* = 5, and the normalized values for *n* = 4 are very close to that for *n* = 5, while the normalized values of IBIC for *n* = 4 is obviously far from *n* = 3 and *n* = 5, which means that the IBIC is more robust and reliable than AIC and MDL for the given case.

Therefore, it can be concluded that all the four source number estimation methods are effective for the given numerical case study, and the eigenvalue decomposition-based method and IBIC are more robust and reliable than AIC and MDL as they have very wide boundaries to accurately determine the source number. However, the eigenvalue decomposition-based method requires a reasonable threshold *γ* normally determined by prior knowledge of the system or experiences, while the other three methods can adaptively estimate the source number.

### 3.2. Source Number Estimation for Weakly Nonlinearly Mixed Signals

In this section, a nonlinearity mixing factor *σ* on the modulation sources is considered in the mixing process, and thus the performances on the nonlinearly mixed signals of all the source number estimation methods are comparatively studied. The mixed signals are composed by the sources in Figure 1 with both a linear superposition and a nonlinear modulation mixing, and their generating functions are shown in Equation (11):

(12) $$X=\left[\begin{array}{l} 0.58s_{2}+0.36s_{2}-0.29s_{3}+0.89s_{4}+\sigma s_{1}s_{2}\\ 0.33s_{1}-0.65s_{2}+0.49s_{3}-0.93s_{4}+\sigma s_{1}s_{3}\\ 0.77s_{1}+0.83s_{2}-0.72s_{3}-0.85s_{4}+\sigma s_{2}s_{3}\\ 0.18s_{1}+0.51s_{2}+0.83s_{3}+0.79s_{4}+\sigma s_{3}s_{2}\\ 0.25s_{1}-0.42s_{2}-0.65s_{3}-0.59s_{4}+\sigma s_{2}s_{1}\\ 0.43s_{1}-0.27s_{2}-0.14s_{3}+0.32s_{4}+\sigma s_{1}s_{3} \end{array}\right]$$

The nonlinearity mixing factor *σ* reveals the modulation effects of the mechanical systems with many sources, and the nonlinear mixing mode is always considered as a technical bottleneck for source separation or source number estimation. Therefore, the factor *σ* is considered as a parameter to test the performances of source number estimation algorithms. It should be noted that the nonlinearity mixing factor *σ* is given an initial value 0.0001 to satisfy the logarithm function in Equations (4), (5) and (9), and the information-based methods are comparatively studied as the nonlinearity mixing factor *σ* increases.

**Figure 4.**
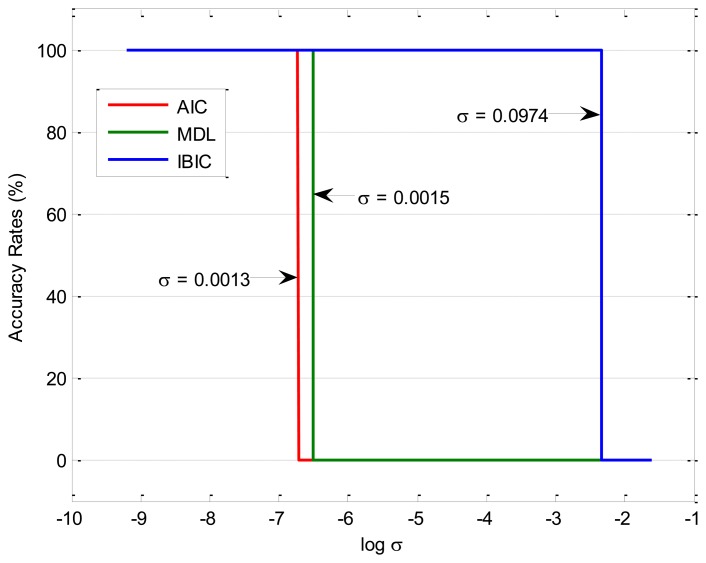
Accuracy rates of information-based source number estimation methods.

**Figure 5.**
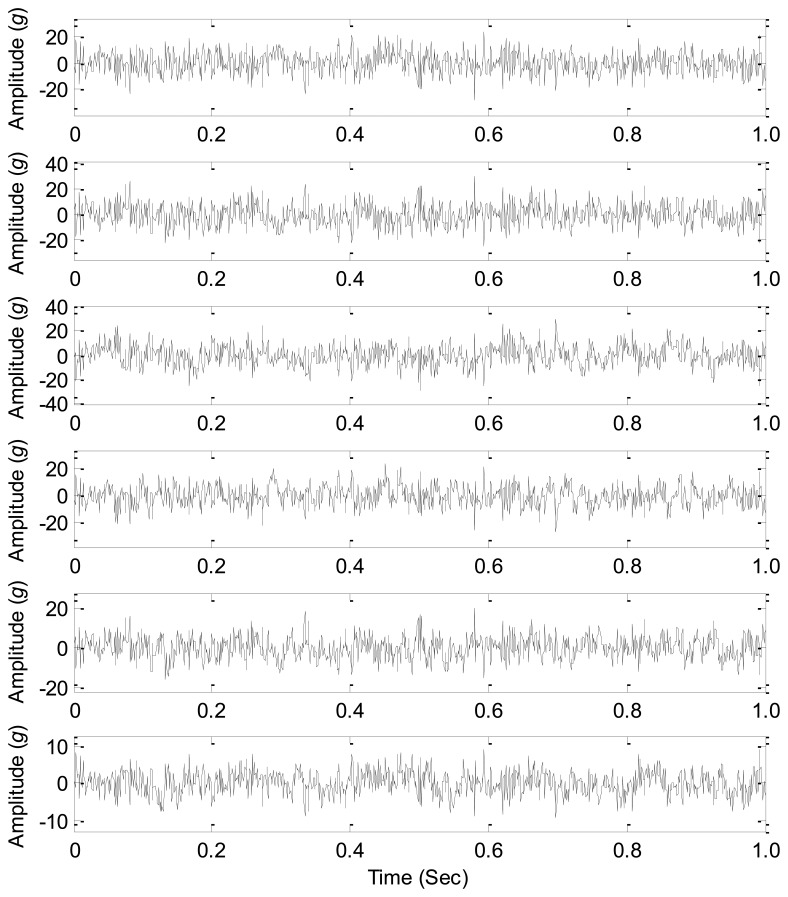
The waveforms of the mixed signals for *σ* = 0.0974.

The accuracy rates of the given three information-based source number estimation methods are displayed in Figure 4, which shows that AIC, MDL and IBIC fail to correctly estimate the source number when the factor *σ* is up to 0.0013, 0.0015, and 0.0974, respectively. The comparative study results indicate that AIC and MDL give similar performances and they are all sensitive to the nonlinearity mixing factor *σ* (*σ* < 0.0013), and MDL performs a little better than AIC toward the nonlinearity mixing factor *σ* but not significantly, while IBIC performs more robustly toward the nonlinearity mixing factor *σ* (*σ* < 0.0974). Therefore, IBIC performances more robustly and reliably toward the nonlinear mixing effects of mechanical systems, and this property guarantees more wide engineering applications of IBIC as most physical systems have nonlinear mixing effects. The waveforms of the mixed signals for *σ* = 0.0974 are shown in Figure 5, which also shows that it cannot directly estimate the source number just from the complicated waveforms.

**Figure 6.**
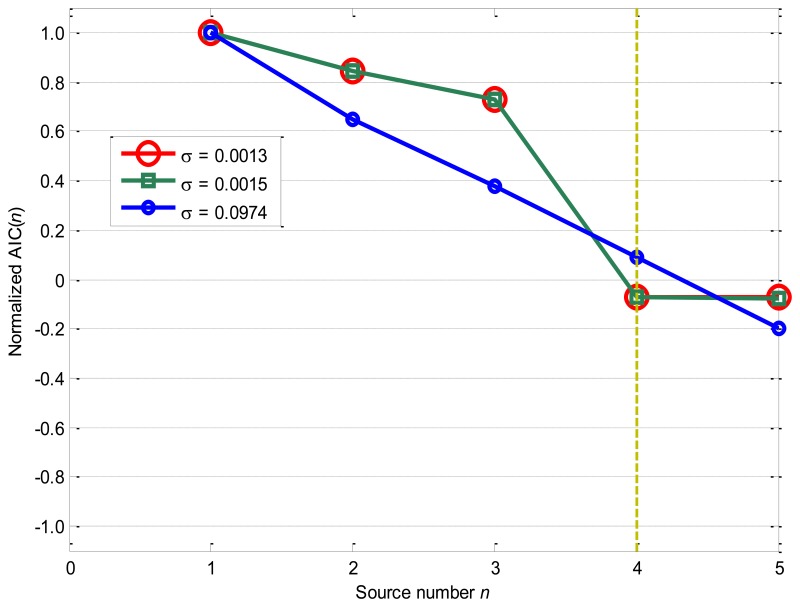
Source number estimation by AIC.

Figure 6 displays the performances of AIC as the nonlinearity mixing factor *σ* equals to 0.0013, 0.0015, and 0.0974: AIC decreases significantly from *n* = 1 to *n* = 5 and obtains the minimum value −0.2014 as *n* = 5 for the *σ* = 0.0974 case. Furthermore, AIC has similar values for the *σ* = 0.0013 and *σ* = 0.0015 cases, and obtains minimum values of −0.0742 and −0.0749 as *n* = 5. However, for the *σ* = 0.0012 case, AIC obtains a minimum value −0.0756 as *n* = 4, while AIC = 0.7322 as *n* = 3 and AIC = −0.0739 as *n* = 5. Therefore, AIC fails to correctly estimate the source number as the nonlinearity mixing factor *σ* increases to 0.0013, and the objective function decreases fast for the *σ* = 0.0974 case, which means that the nonlinear modulation effects influence the accuracy rates of AIC greatly.

**Figure 7.**
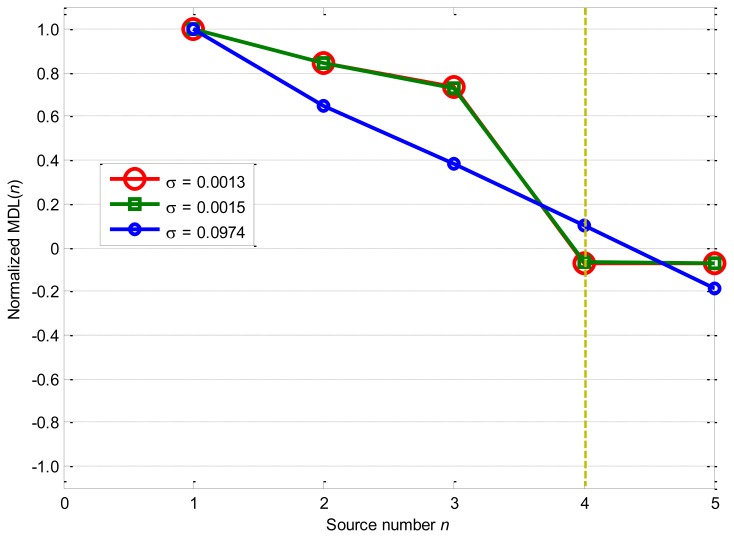
Source number estimation by MDL.

Figure 7 displays the performances of MDL as the nonlinearity mixing factor *σ* equals 0.0013, 0.0015, and 0.0974: MDL decreases significantly from *n* = 1 to *n* = 5 and obtains the minimum value −0.1898 as *n* = 5 for *σ* = 0.0974 case.

For the *σ* = 0.0013 case, MDL obtains the minimum value −0.0703 as *n* = 4, which follows this rule until σ = 0.0015 where MDL begins to have the minimum values as *n* = 5. Comparing Figure 6 with Figure 7, it can be seen that AIC and MDL perform closely for all these three cases, and the only difference is that MDL is a little more robust to the nonlinearity mixing factor *σ* than AIC.

**Figure 8.**
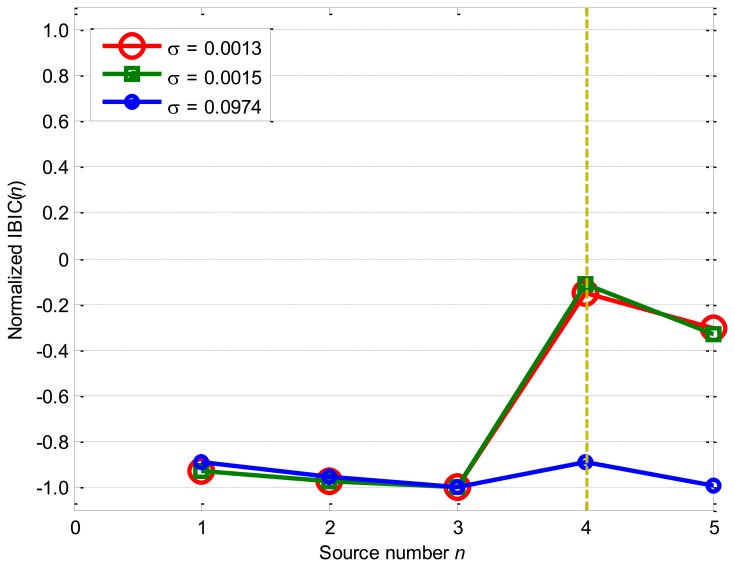
Source number estimation by IBIC.

Figure 8 displays the performances of IBIC as the nonlinearity mixing factor *σ* equals to 0.0013, 0.0015, and 0.0974: IBIC obtains the maximum values −0.1490 and −0.1106 as *n* = 4 for *σ* = 0.0013 and *σ* = 0.0015 cases, and begins to have the maximum value −0.8935 as *n* = 1 for the *σ* = 0.0974 case.

Furthermore, the maximum values of IBIC for *σ* = 0.0013 and *σ* = 0.0015 cases are obviously far from their neighbors, which guarantees a more accurate estimation of the source number. However, IBIC values close to each other for *σ* = 0.0974 case, which means that the maximum values of IBIC are difficult to be determined and IBIC becomes less robustly and reliably.

Table 2 displays the eigenvalues of covariance matrix with different factors *σ*: the related eigenvalues are very close to each other for *σ* = 0.000, 0.0013, and 0.0015 cases, while *λ*_5_ is up to 0.9791 for the *σ* = 0.0974 case. From the definition of eigenvalue decomposition-based source number estimation method, *λ*_5_ = 0.0002 causes AIC failure for the *σ* = 0.0013 case, and *λ*_5_ = 0.0003 causes MDL failure for the *σ* = 0.0015 case. However, IBIC begins to fail for the *σ* = 0.0974 case, which also indicates that IBIC is much more robust to the modulation effects than AIC and MDL. Furthermore, the distributions of all the eigenvalues show that it is not difficult to set a threshold *γ* = (0.9791, 4.37) for the eigenvalue decomposition-based source number estimation method. However, normally it is very difficult to set a reasonable *γ* without any prior knowledge of the sources and their distributions.

Therefore, it can be concluded that the eigenvalue decomposition-based source number estimation method is difficult to carry out without any prior knowledge of the sources, while the information-based methods can adaptively and accurately estimate the source number for the linear superposition cases. However, for the cases with nonlinear modulation effects, IBIC performs more robustly and reliably than AIC and MDL, which reveals more wide engineering applications of IBIC.

## 4. Experimental Study

**Figure 9.**
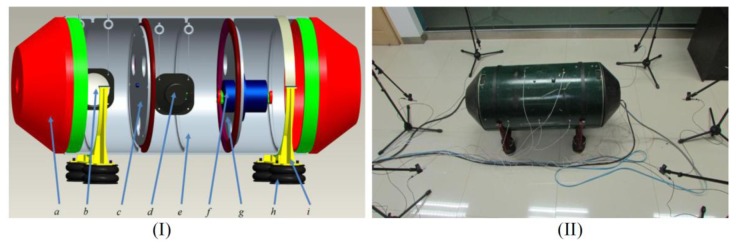
The structure (I) and photo (II) of the test-bed: (**a**) End cover. (**b**) Loudspeaker I. (**c**) Left clapboard. (**d**) Loudspeaker II. (**e**) Shell. (**f**) Motor. (**g**) Right clapboard. (**h**) Rubber springs. (**i**) Supports.

In general, it is difficult to directly measure the source signals in most mechanical systems due to the limited accessibility, and thus signal processing is often required to separate and recover the source information from the mixed signals normally measured by remote sensors. Then, these separated source signals can be used for further purposes such as a condition monitoring and a fault diagnosis of mechanical systems. However, a source number estimation from the measured and mixed signals should be carried out for a prior knowledge to source separation or complexity analysis of the systems. In this section, we apply the source number estimation methods mentioned above to a mechanical system shown in Figure 9 to demonstrate and benchmark their performance on mechanical systems.

### 4.1. Introduction of the Test Bed

Aiming at vibration and noise source number estimation for mechanical systems, this study designs a test bed based on a shell structure, which is composed by an end cover, a shell, clapboards, and supports. The whole test bed is supported by four rubber air springs, which can reduce the influences of environmental noises. There are three sound sources: two of them are loudspeakers controlled by the signal generators, and the other one is a motor controlled by the frequency converter. The structure and photo of the test bed are shown in Figure 9. Since vibration and noises of thin shell structures can approximately be governed by linear differential equations [30], we consider the test bed with linear but weakly nonlinear features.

**Figure 10.**
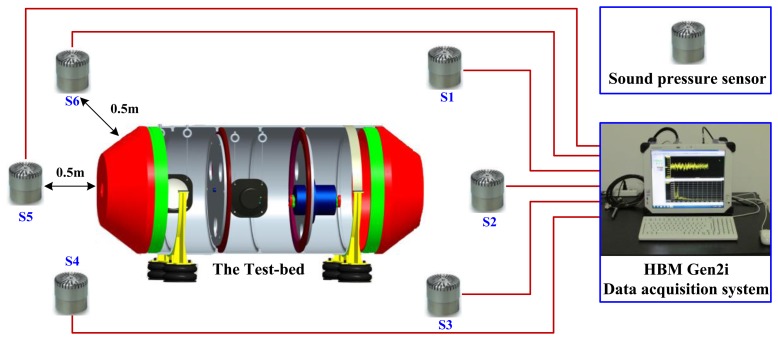
The measuring system of the test bed.

Six sound pressure sensors are used to measure the sound information, and they are installed in different directions of the test bed with a distance of 0.5 m. A HBM Gen2i data acquisition system is applied to collect the sound data from these six sensors. The framework of the measuring system is shown in Figure 10, and the testing parameters are listed in Table 3.

### 4.2. Source Number Estimation

**Figure 11.**
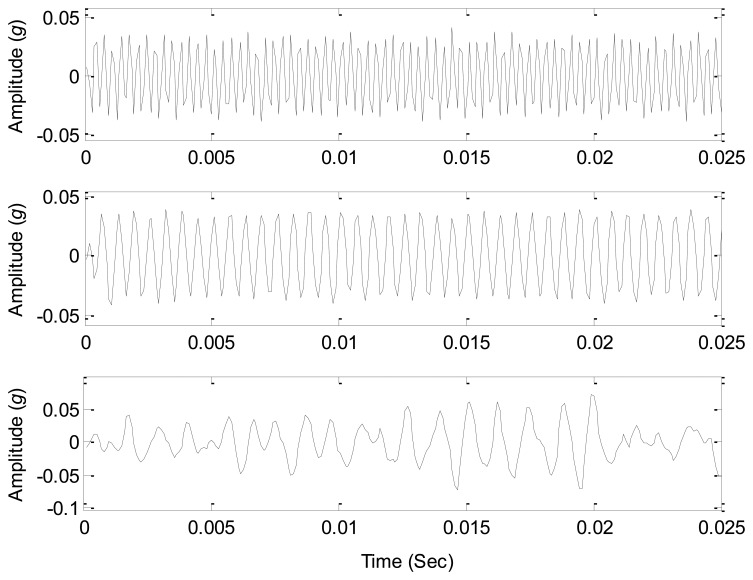
Waveforms of the source signals.

**Figure 12.**
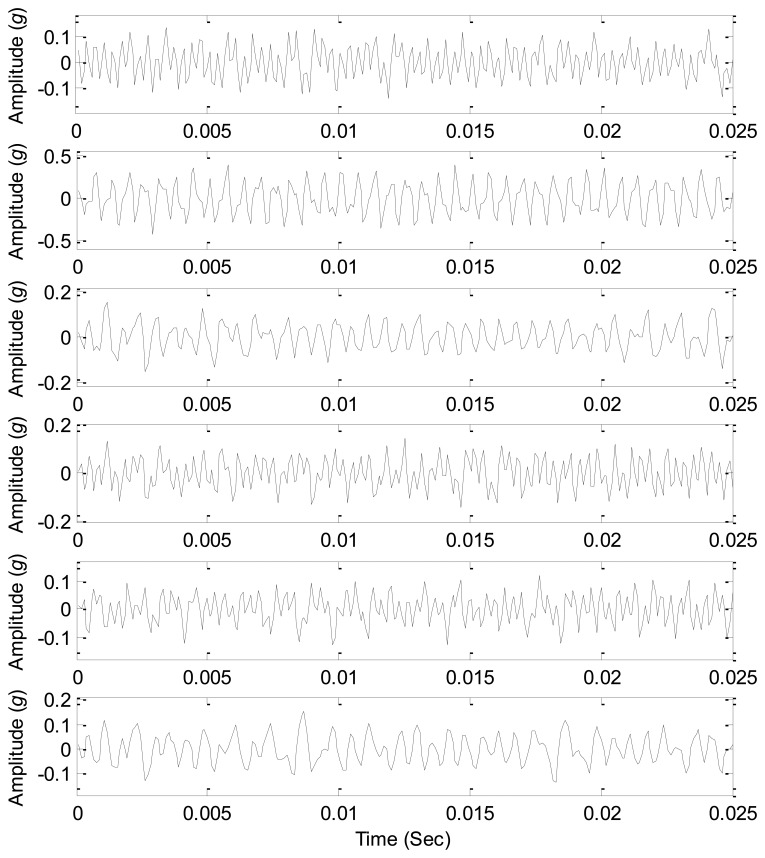
Waveforms of the mixed signals.

The sound source signals are measured with just one source working at the parameters given in Table 4, and thus three sound sources can be measured as the references to test the source number estimation methods. The waveforms of the source signals are shown in Figure 11, which clearly displays the periodic features of the source signals. As all the three sources are working together, the signals from all the sound pressure sensors around are the mixed signals, and their waveforms are shown in Figure 12. Obviously it is very difficult to correctly estimate the source information from the mixed signals due to complicated waveforms.

Both eigenvalue decomposition-based source number estimation method and information-based source number estimation methods are applied to estimate the sound source number of the given test bed. The eigenvalues of the covariance matrix for the mixed signals in different parameters are shown in Table 4. Obviously, it is difficult to determine the source number just based on the distributions of the eigenvalues as *λ*_4_, *λ*_5_ and *λ*_6_ are very close to each other.

**Figure 13.**
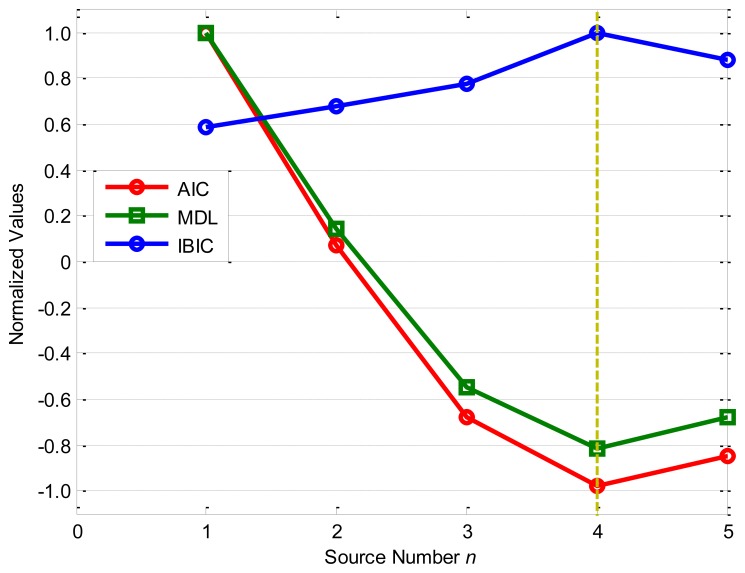
Source number estimation by information-based methods.

The results of source number estimation by information-based methods are shown in Figure 13, which clearly shows that AIC and MDL obtain minimum values −0.9806 and −0.8171 as *n* = 4, while IBIC also obtains the maximum value 1.0000 as *n* = 4. Therefore, all the information-based source number estimation methods provide an estimated source number 4 for the given experimental study. However, there are only three sources in the test bed. The inconsistent results confuse us seriously but we can provide more convincing evidences based on a source separation by independent component analysis (known as ICA).

**Figure 14.**
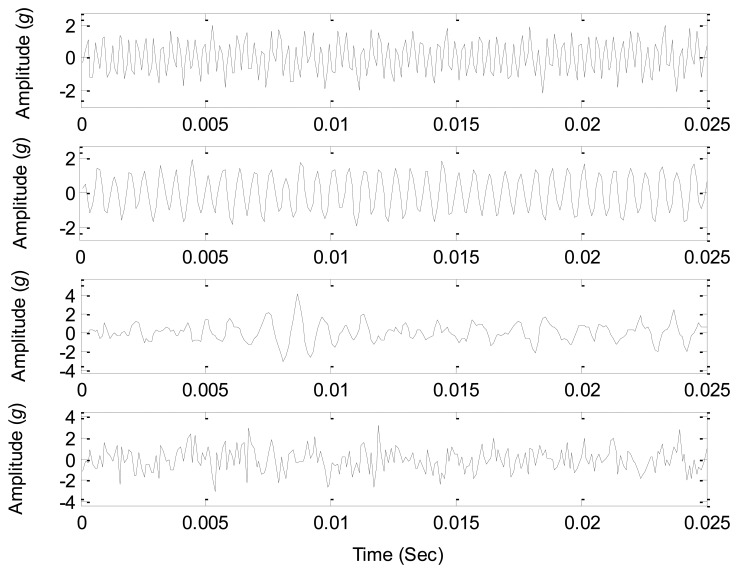
The waveforms of the separated components by fast ICA algorithm.

The fast ICA algorithm [6,31] is applied to source separation from the given six mixed signals, and the parameter of the source number is given an initial value 4. Thus we obtain four independent components from the mixed signals, and their waveforms are shown in Figure 14, which displays that the waveforms of three separated components are similar to that of the related source signals in Figure 11, and the other separated signal has complex waveforms and is obviously different from the given source signals.

**Figure 15.**
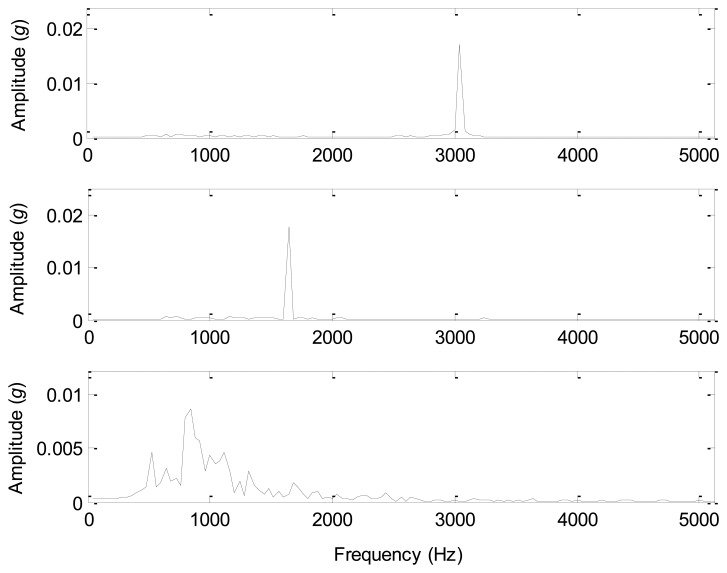
The spectra of the source signals.

**Figure 16.**
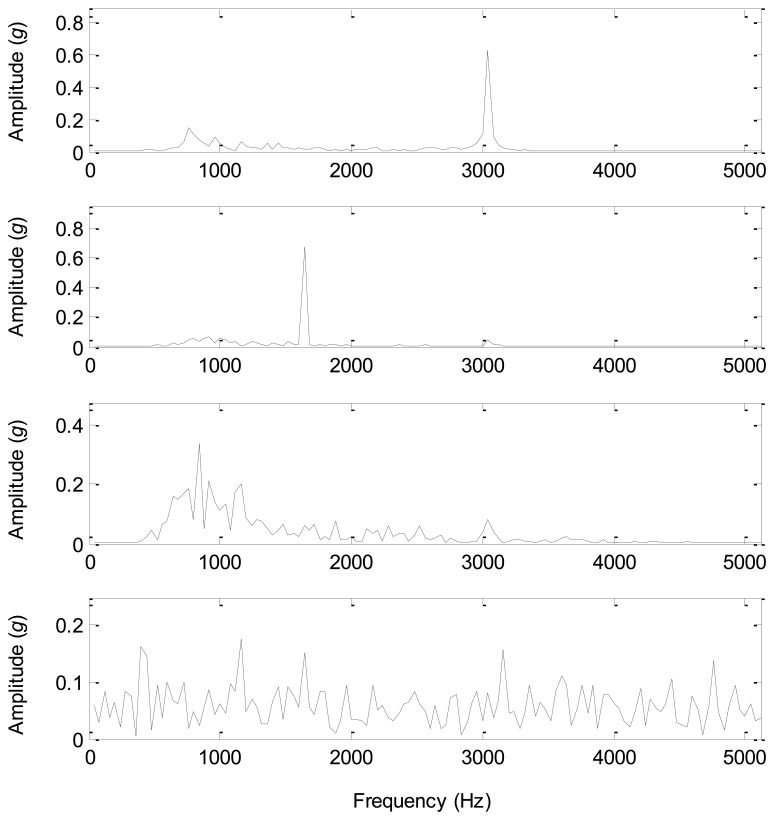
The spectra of the separated components.

The spectra of the source signals and the separated components are displayed in Figure 15 and Figure 16. Comparing Figure 15 with Figure 16, the spectrum of the separated component 1 is similar to that of the source signal 1 with a same significant frequency of 3,000 Hz, and the spectrum of the separated component 2 is similar to that of the source signal 2 with a same significant frequency of 1,600 Hz. Furthermore, the spectrum of the separated component 3 is close to that of the source signal 3, and the major frequency energy is contained in 500–1,500 Hz. However, the spectrum of the separated component 4 is very complicated from 0 to 5,000 Hz, which is similar to the spectra of white noises. Therefore, the separated component 4 that we consider as an environmental noise has a considerable energy compared with the other three sources, and causes the source number to be 4 rather than 3. After the source separation and spectral analysis, the correct source number of the given test bed should be 4 rather than 3, which provides convincing evidence for the accuracy of all the information-based methods.

In general, like the similar values of the eigenvalues, it is very difficult for the eigenvalue decomposition-based method to accurately and robustly estimate the source number without any prior information about the sources. However, all the information-based methods correctly and adaptively estimate the source number of the test bed, which further reveals that the mixing mode of the sound sources tends to be a linear superposition, and thus guarantees that the information-based methods are effective to the sound signals.

## 5. Conclusions

This paper investigates both eigenvalue decomposition-based and information-based source number estimation methods, and improves BIC as IBIC to make it more efficient and easier to calculate. Furthermore, their performances on nonlinear modulation effects of mechanical systems are studied comparatively with numerical case studies and experimental studies.

In the numerical case study with a linear superposition case, the eigenvalue decomposition-based method has a wide band to determine the threshold *γ* ∈ (0, 4.42), and all the three information-based methods accurately estimate the source number. Furthermore, IBIC performs more robustly and effectively as obvious differences of objective functions. For the weakly nonlinearly mixing case, AIC and MDL are very sensitive to the modulation factor *σ* and become inaccurate with a very small *σ*, while IBIC performs more robustly and reliably toward *σ*, which means that IBIC is more effective to mechanical systems. In the experimental studies, a test bed with three sound sources is constructed to test the performances of the above methods. The eigenvalue decomposition-based method is difficult to determine the threshold as the last three eigenvalues are very close to each other. However, all the three information-based methods obtain extremum values as *n* = 4. Using a source separation, four independent components are extracted from the mixed signals, and the other source which has a considerable energy with the given three sources is considered as an environmental noise. Therefore, all the information-based methods are effectively to sound source number estimation for the given test bed.

Generally, IBIC performs more robustly and reliably toward the nonlinear modulation effects than AIC and MDL, while eigenvalue decomposition-based methods normally require prior information about the sources, and becomes confused when the eigenvalues are very close to each other. Furthermore, the results of information-based methods for the test bed also indicate that the mixing mode of the sound sources tends to be a linear superposition. This study can benefit for model order selection, complexity analysis of a system, and applications of source separation to mechanical systems for condition monitoring and fault diagnosis purposes.

## Figures and Tables

**Table 1.** The eigenvalues of the covariance matrix for the mixed signals.

**Eigenvalues**	*λ*_1_	*λ*_2_	*λ*_3_	*λ*_4_	*λ*_5_	*λ*_6_
Values	281.31	31.21	10.43	4.42	0.00	0.00

**Table 2.** Eigenvalues of covariance matrix with different factor *σ*.

**Eigenvalues**	*λ*_1_	*λ*_2_	*λ*_3_	*λ*_4_	*λ*_5_	*λ*_6_
*σ* = 0.0000	281.31	31.21	10.43	4.42	0.0000	0.0000
*σ* = 0.0013	289.56	31.25	10.48	4.37	0.0002	0.0000
*σ* = 0.0015	271.56	31.27	10.41	4.37	0.0003	0.0001
*σ* = 0.0974	280.06	31.63	12.66	5.26	0.9791	0.0206

**Table 3.** The testing parameters of the measuring system.

**Parameters**	**Values and Units**
Sound pressure sensors	6
HBM Gen2i Data acquisition system	1
Sampling frequency	10,240 Hz
Sampling length	10 s
Frequency of Loudspeaker I with sine wave	*f*_1_ = 1,600 Hz
Frequency of Loudspeaker II with triangle wave	*f*_2_ = 3,000 Hz
Rotational speed of motor	900 r/min (*f*_3_ = 15Hz)

**Table 4.** The eigenvalues of the covariance matrix for the mixed signals.

**Eigenvalues**	*λ*_1_	*λ*_2_	*λ*_3_	*λ*_4_	*λ*_5_	*λ*_6_
*f*_1_ = 3000 *f*_2_ = 1600 *f*_3_ = 15	0.0306	0.0076	0.0047	0.0022	0.0010	0.0009
*f*_1_ = 3000 *f*_2_ = 1600 *f*_3_ = 20	0.0386	0.0252	0.0097	0.0067	0.0049	0.0038
*f*_1_ = 3000 *f*_2_ = 1600 *f*_3_ = 25	0.0450	0.0121	0.0116	0.0082	0.0061	0.0045
